# Minimal Diversity of Drug-Resistant *Mycobacterium tuberculosis* Strains, South Africa^1^

**DOI:** 10.3201/eid2003.131083

**Published:** 2014-03

**Authors:** Neel R. Gandhi, James C.M. Brust, Prashini Moodley, Darren Weissman, Moonseong Heo, Yuming Ning, Anthony P. Moll, Gerald H. Friedland, A. Willem Sturm, N. Sarita Shah

**Affiliations:** Albert Einstein College of Medicine, Bronx, New York, USA (N.R. Gandhi, J.C.M. Brust, D. Weissman, M. Heo, Y. Ning, N.S. Shah);; Rollins School of Public Health, Emory University, Atlanta, Georgia, USA (N.R. Gandhi);; Nelson R. Mandela School of Medicine, University of KwaZulu-Natal, Durban, South Africa (P. Moodley, A.W. Sturm);; *Philanjalo*, Tugela Ferry, South Africa (A.P. Moll); Church of Scotland Hospital, Tugela Ferry (A.P. Moll);; Yale University School of Medicine, New Haven, Connecticut, USA (G.H. Friedland)

**Keywords:** Mycobacterium tuberculosis, drug resistance, transmission, genotype, South Africa, HIV, bacteria, tuberculosis, tuberculosis and other mycobacteria, antimicrobial resistance

## Abstract

Multidrug- (MDR) and extensively drug-resistant tuberculosis (XDR TB) are commonly associated with Beijing strains. However, in KwaZulu-Natal, South Africa, which has among the highest incidence and mortality for MDR and XDR TB, data suggest that non-Beijing strains are driving the epidemic. We conducted a retrospective study to characterize the strain prevalence among drug-susceptible, MDR, and XDR TB cases and determine associations between strain type and survival. Among 297 isolates from 2005–2006, 49 spoligotype patterns were found. Predominant strains were Beijing (ST1) among drug-susceptible isolates (27%), S/Quebec (ST34) in MDR TB (34%) and LAM4/KZN (ST60) in XDR TB (89%). More than 90% of patients were HIV co-infected. MDR TB and XDR TB were independently associated with mortality, but TB strain type was not. We conclude that, although Beijing strain was common among drug-susceptible TB, other strains predominated among MDR TB and XDR TB cases. Drug-resistance was a stronger predictor of survival than strain type.

Drug-resistant tuberculosis (TB) has emerged as a substantial threat to advances in global TB control over the past several decades (*1*). Worldwide, an estimated 630,000 cases of multidrug-resistant (MDR) TB occurred in 2011, and extensively drug-resistant (XDR) TB has now been reported in 84 countries (*2*). MDR TB and XDR TB are each associated with very high mortality rates (*3*), and their transmission—both in community and health care settings—remains an ongoing challenge in resource-limited settings and in countries with high rates of HIV co-infection.

In South Africa, the incidence of MDR TB has increased 5-fold since 2002 (*2*,*4*). MDR TB treatment is now estimated to consume more than half of the budget allocated for TB control in South Africa (*5*). The emergence of XDR TB, and its associated high mortality rates, have further underscored the need for clarifying the factors driving the drug-resistant TB epidemic to better focus control efforts (*3*,*6*,*7*).

Drug-resistant TB is generally considered a human-made phenomenon that occurs when inadequate TB treatment creates selection pressure for the emergence of drug-resistant Mycobacterium tuberculosis subpopulations (acquired resistance) (*1*). Researchers initially believed that the mutations causing drug resistance would exert a “fitness cost,” rendering those strains too weak to be transmitted (*8*,*9*). Nonetheless, transmission of drug-resistant TB strains has now been well-documented (*10*–*13*), and laboratory studies have shown that clinical strains may have minimal fitness costs or even none (*14*). Emerging data suggest that most MDR TB and XDR TB cases in South Africa and worldwide are likely caused by primary transmission of drug-resistant strains (*2*,*15*–*19*).

Although the *M*. *tuberculosis* W/Beijing strain family has been described among cases of drug-susceptible, MDR TB, and XDR TB in South Africa, numerous other strain types have also been identified (*20*,*21*). Little is known about the transmissibility and virulence of *M. tuberculosis* strains aside from the W/Beijing strain family (*22*,*23*). In the Eastern Cape and Western Cape Provinces of South Africa, strains from the W/Beijing family have most often been associated with transmission of drug-resistant TB (*24*–*27*). At our study site in KwaZulu-Natal Province, however, the LAM4/KZN strain type has predominated among MDR TB and XDR TB cases and has been linked to nosocomial transmission and high mortality rates (*3*,*16*,*17*,*28*,*29*). This strain is a member of the Euro-American strain family and was first described in this region in 1994, evolving into an increasingly resistant phenotype over time (*29*).

The reasons for why the LAM4/KZN strain is prominent in KwaZulu-Natal Province, rather than the Beijing strain, which is seen globally and in other parts of South Africa, is unclear. Moreover, it is unknown whether the higher mortality among patients with MDR TB and XDR TB in KwaZulu-Natal can be explained, in part, by a difference in genotypic prevalence and associated differences in strain virulence (*3*,*6*,*7*,*28*). In this study, we sought to characterize the genotypic diversity of *M. tuberculosis* strains among isolates causing drug-susceptible TB, MDR TB, and XDR TB in KwaZulu-Natal Province, South Africa. We also examined the relationship between *M. tuberculosis* strain, drug resistance, and patient survival.

## Methods

### Study Design and Population

We performed a retrospective study of patients who had received diagnoses of drug-susceptible TB, MDR TB, and XDR TB in Tugela Ferry, KwaZulu-Natal Province, from January 1, 2005, through December 31, 2006. Patients were eligible if their medical records and an *M. tuberculosis* isolate were available for analysis (*30*). The study was approved by the institutional review boards at the University of KwaZulu-Natal, Albert Einstein College of Medicine, and Yale University, and by the KwaZulu-Natal Department of Health. 

### Setting

Tugela Ferry is a town situated in a rural district with a population of 200,000 persons. A single, 355-bed government district hospital provides inpatient care. In 2006, the incidence of drug-susceptible TB was 1,100 cases/100,000 population, and MDR TB incidence was 119 per 100,000 persons (*3*). More than 80% of TB case-patients were co-infected with HIV, and the antenatal HIV prevalence was 37%. 

Since June 2005, after a large cluster of MDR TB and XDR TB cases were discovered in Tugela Ferry, clinicians there have been encouraged to evaluate all persons with suspected TB by ordering mycobacterial culture and drug-susceptibility testing (DST) in addition to smear microscopy. This practice differed from South African national policy, which recommended culture and DST be requested only when patients were experiencing treatment failure or receiving re-treatment (*31*). Detailed methods regarding sputum collection, microscopy, culture, and DST have been previously described (*28*).

All new TB patients began empiric first-line therapy (administration of isoniazid, rifampin, ethambutol, and pyrazinamide for 2 months, followed by administration of isoniazid and rifampin for 4 months), whereas re-treatment patients began a standard category II regimen (*31*). Second-line therapy for drug-resistant TB was not available at the Tugela Ferry hospital. Patients with confirmed MDR TB or XDR TB were transferred to a referral hospital in Durban for treatment of drug-resistant TB. The average time from sputum collection to transfer was 111 days for XDR TB patients (*4*), during which time patients remained on the inpatient wards receiving first-line TB therapy.

Upon transfer to the TB referral hospital, MDR TB patients received a standardized treatment regimen of kanamycin, ofloxacin, ethionamide, ethambutol, pyrazinamide, and terizidone for at least 4 months, followed by the same regimen without kanamycin for an additional 18 months. XDR TB patients received the same regimen until 2007, when capreomycin and para-aminosalicylic acid became available in South Africa and replaced kanamycin and ofloxacin. Third-line TB drugs and surgical treatment were not routinely used at the time of this study.

### Medical Record Review and Genotyping

Medical records were reviewed for the following patient characteristics: sex, age, HIV history (HIV status, CD4 count, viral load, receipt of antiretroviral therapy), TB history (acid-fast bacilli smear status, presence of extrapulmonary TB, previous treatment episodes), previous hospitalizations, whether patients were referred for second-line TB therapy, and survival. TB isolates underwent spoligotyping using a commercially available kit. Spoligotype patterns were classified according to the 4th International Spoligotyping Database.

### Analysis 

We described spoligotype distribution among drug-susceptible TB, MDR TB, and XDR TB isolates by using simple frequencies and proportions overall, and stratified by HIV status. Duplicate isolates from the same patient were included in the study only if they differed in drug resistance pattern or spoligotype. To provide a comprehensive description of the genotypic diversity found, each isolate was reported in the respective drug resistance or spoligotype groups. Thus, the number of isolates exceeds the number of patients in the description of spoligotype distributions.

We tested the association between spoligotype pattern and survival among MDR TB and XDR TB patients by bivariate and multivariable analysis, using product limit estimates and Cox proportional hazards analysis. To account for patients with multiple isolates of differing drug resistance or spoligotype pattern, we analyzed drug resistance group and spoligotype as time-dependent covariates. When 2 isolates were collected from a single subject on the same day, the bivariate and multivariate analyses were first run by using the less-resistant isolate and then by using the more- resistant isolate for sensitivity analysis. The direction and magnitude of the results did not change regardless of the technique (data not shown). Additionally, to account for missing CD4 counts for multivariable analysis, we performed multiple imputation using a Markov Chain Monte Carlo method as previously described (*30*).

## Results

There were 227 patients who contributed 297 TB isolates for this study. Eighty-six (38%) patients had drug-susceptible TB, 67 (30%) had MDR TB; and 74 (33%) had XDR-TB. The median age was 33–34 years among patients in each drug resistance group (Table 1). More than 90% of patients were HIV co-infected, with a median CD4 count of <100 cells/mm^3^. The majority of patients had positive acid-fast bacilli smear results, and nearly one-quarter had both extrapulmonary and pulmonary TB disease. Approximately 70% of patients with MDR TB or XDR TB had previously received TB treatment, whereas 34% of patients with drug-susceptible TB had been previously treated. Recent hospitalization was also more common among patients with MDR TB or XDR TB (52% and 59%, respectively), than among those with drug-susceptible TB (21%, p<0.0001).

**Table 1:** Demographic and clinical characteristics of patients with drug-susceptible, MDR TB, and XDR TB, Tugela Ferry, KwaZulu-Natal Province, South Africa 2005–2006*

Characteristic	Drug-susceptible TB	MDR TB	XDR TB
Total no.	86	67	74
Female sex	31 (36)	34 (51)	38 (51)
Age, y, median (IQR)	34 (29–42)	34 (29–43)	33 (29–40)
Tested for HIV	68 (85)	49 (73)	63 (79)
HIV-positive†	61 (90)	45 (92)	63 (100)
CD4 available at diagnosis	33 (54)	30 (67)	32 (51)
Median cells/mm^3^ (IQR)	74 (29–129)	85.5 (47–217)	94 (46.5–169)
Viral load available at diagnosis	20 (33)	11 (24)	15 (24)
Median copies/mL (IQR)	110,000 (23,000–570,000)	120,000 (2,000–200,000)	71,000 (89–530,000)
Receiving ARV therapy at diagnosis	19 (31)	12 (27)	18 (29)
Sputum smear result available	80 (93)	65 (97)	72 (97)
Positive	45 (56)	43 (66)	50 (69)
Presence of extrapulmonary TB	20 (23)	17 (25)	22 (30)
Previous TB treatment†			
Any	29 (34)	47 (70)	53 (72)
Previous hospitalization†			
Past 2 y	18 (21)	35 (52)	44 (59)

*N = 227; values indicate no. (%) unless otherwise noted. MDR TB, multidrug-resistant tuberculosis; XDR TB, extensively drug-resistant TB; IQR, interquartile range; ARV, antiretroviral. †p<0.05.

### Strain Diversity

**Figure 1:**
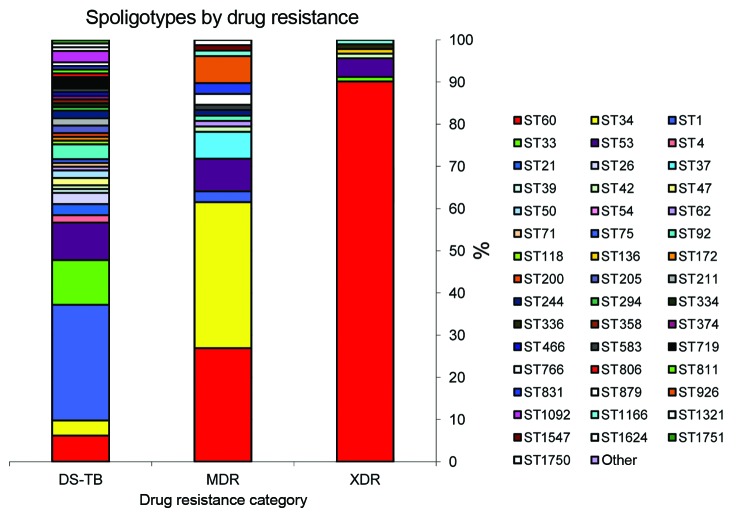
Distribution of spoligotype patterns among drug-susceptible (DS-TB), multidrug-resistant (MDR) tuberculosis and extensively drug-resistant (XDR) cases in Tugela Ferry, KwaZulu-Natal Province, South Africa, 2005–2006. *Does not include 11 isolates with unknown drug-susceptibility test results.

Among the 297 isolates analyzed, we found 49 different spoligotype patterns (Table 2). The distribution of spoligotypes varied between drug-resistance categories; as drug resistance increased, strain diversity decreased (p<0.0001 for trend) (Figure 1).

**Table 2:** Spoligotype patterns of *Mycobacterium tuberculosis* isolates from patients in Tugela Ferry, KwaZulu-Natal Province, South Africa, 2005–2006*†‡

Lineage	Shared type	International family	Octal code	DS TB strains, n = 115	MDR TB strains, n = 79	XDR TB strains, n = 92
**Beijing**	**1**	**Beijing**	**000000000003771**	**31**	**2**	**0**
LAM	4	LAM3/S	000000007760771	2	0	0
	33	LAM3	776177607760771	12	0	1
	42	LAM9	777777607760771	1	1	1
	**60**	**LAM4**	**777777607760731**	**7**	**21**	**82**
	211	LAM3	776137607760771	2	0	0
	811	LAM4	777777604060731	1	0	0
	1321	LAM1-LAM4	677777607760731	1	0	0
	1624	LAM3-LAM6	776177607560771	1	0	0
	1750	LAM4	777767607760731	0	1	0
S family	**34**	**S**	**776377777760771**	**4**	**27**	**0**
	466	S	776377377760771	1	0	0
	831	S	776367777760771	1	2	0
T family	37	T3	777737777760771	0	5	0
	39	T4-CEU1	777777347760471	1	0	0
	53	T1	777777777760771	10	6	4
	118	T2	777767777760771	1	0	0
	136	T1	777603405760471	0	0	1
	205	T1	737777777760771	2	0	0
	244	T1	777777777760601	2	1	0
	334	T1	577777777760771	1	0	0
	358	T1	717777777760771	1	0	0
	719	T1	776177407760771	3	0	0
	766	T1	777761007760771	0	2	0
	879	T1	777767777760671	1	0	0
	926	T1	773777777760771	0	5	0
	1166	T1	777377777760771	0	1	1
	1547	T3	777727777760771	0	1	0
X family	200	X3	700076777760700	1	0	0
	336	X1	777776777760731	0	0	1
	1751	X3	700066777760771	1	0	0
Haarlem	47	H1	777777774020771	2	0	0
	50	H3	777777777720771	2	0	0
	62	H1-variant1	777777774020731	1	1	0
	75	H3	777767777720771	1	0	0
	294	H3	577777777720771	1	0	0
Other	21	CAS1-Kili	703377400001771	3	0	0
	26	CAS1-Delhi	703777740003771	3	0	0
	71	EAI-undefined	776337777760771	1	0	0
	172	U	777777777740771	1	0	0
	374	U	777777776000771	1	0	0
	583	MANU2	777737777763771	1	1	0
	806	EAI1-SOM	757777777413731	1	0	0
	1092	CAS	702777740003771	3	0	0
*DS TB, drug-susceptible tuberculosis; MDR TB, multidrug-resistant TB; XDR TB, extensively drug-resistant TB. Boldface indicates the most common strain for each resistance group. †Does not include 11 isolates with unknown drug-susceptibility test results. ‡See online Technical Appendix (wwwnc.cdc.gov/EID/article/20/3/13-1083-Techapp1.pdf) for all spoligotype patterns in binary format.						

Thirty-eight different spoligotype patterns were identified among the 115 drug-susceptible TB isolates (Table 2; Figure 1). W/Beijing strain (ST1) was most common, accounting for 27% (n = 31) of isolates, followed by ST33 (10%; n = 12). The remaining 72 isolates were distributed over 36 unique spoligotype patterns (Table 2; Figure 1).

Three predominant spoligotype patterns were found among the 79 MDR TB isolates (ST34, ST60, and ST53) and accounted for 69% (n = 54) of isolates. The S/Québec family (ST34) was most common (n = 27, 34%), followed by the LAM4/KZN family (ST60, n = 21, 27%) and the T1 family (ST53, n = 6, 8%). The Beijing family (ST1) occurred in 2 (3%) MDR TB isolates. The remaining 23 MDR TB isolates exhibited 13 different spoligotype patterns (ST37, ST42, ST62, ST90, ST92, ST244, ST583, ST766, ST831, ST926, ST1166, ST1547, and ST1750).

The least genotypic diversity was seen among XDR TB isolates: 89% (n = 82) of isolates identified as LAM4/KZN strain (ST60). The T1 strain (ST53) was seen in 4 (4%) isolates. The remaining 6 isolates each had distinct spoligotype patterns (ST33, ST42, ST90, ST136, ST336, and ST1166). None of the XDR TB strains were from the Beijing family.

### Mortality

**Figure 2:**
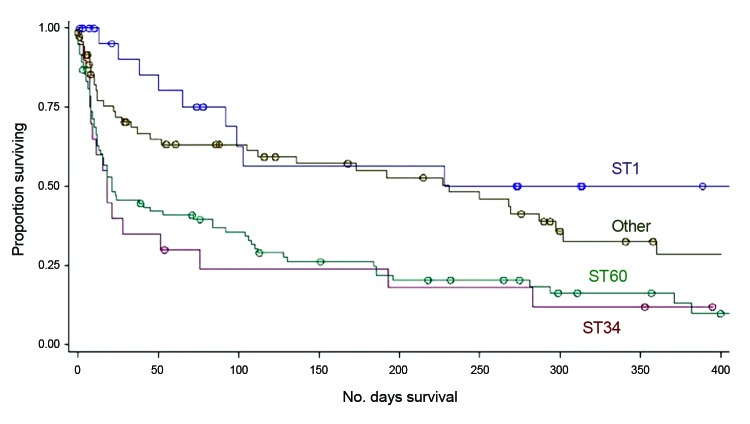
Kaplan-Meier survival distribution, from date of initial sputum collection, stratified by spoligotype (ST1 [Beijing], ST60 [LAM4/KZN], ST34 [S/Quebec] and all others).

Overall, 148 (65%) patients died within 1 year of receiving a diagnosis of drug-resistant TB. Risk factors for death have been described previously and included drug resistance group, positive acid-fast bacilli smear, low CD4 count, presence of extrapulmonary disease, and recent hospitalization (*30*). In this study, mortality was additionally found to be associated with TB strain genotype in bivariate analysis: ST60 (KZN strain) and ST34 (Québec) were both associated with increased mortality, whereas ST1 (Beijing strain) was not (Figure 2).

According to multivariate analysis, however, MDR TB and XDR TB remained independently associated with mortality (MDR hazard ratio [HR] 3.37, p<0.0001; and XDR HR 6.75, p<0.0001), but TB strain type did not (Table 3). Low CD4 count, presence of extrapulmonary TB, and recent hospitalization also remained independently associated with mortality.

**Table 3:** Association of clinical factors and TB genotype with mortality based on Cox proportional hazards analysis, Tugela Ferry, KwaZulu-Natal Province, South Africa, 2005–2006*†

Category	Unadjusted hazard ratio	p value	Adjusted hazard ratio	p value
Spoligotype (ST) pattern	Ref		Ref	–
ST60 (KZN)	2.78	<0.0001	0.14	0.60
ST34 (Québec)	2.48	0.002	0.65	0.07
ST1 (Beijing)	0.66	0.26	0.72	0.42
All other ST types	Ref		Ref	
DST group: drug-susceptible	Ref		Ref	
MDR	3.37	<0.0001	3.33	<0.0001
XDR	5.78	<0.0001	4.68	<0.0001
Positive sputum smear			1.32	0.20
Extrapulmonary TB			1.67	0.02
CD4 count				
<50 cells/mm^3^			2.46	0.007
51–200 cells/mm^3^			1.41	0.30
>200 cells/mm^3^	Ref		Ref	–
Recent hospitalization			2.81	<0.0001

*Ref, referent; KZN, KwaZulu-Natal DST; drug susceptibility testing; MDR, multidrug-resistant, XDR, extensively drug resistant. †If a patient had differing isolates on the same day, the less resistant of the 2 was used for these analyses. Results did not change when the more resistant isolate was used (data not shown).

## Discussion

We examined the genotypic diversity among *M. tuberculosis* strains causing drug-susceptible TB, MDR TB and XDR TB strains from 2005–2006 to better understand the predominance of the LAM4/KZN strain among XDR TB cases in Tugela Ferry, KwaZulu-Natal. We found that a wide variety of TB strains existed among patients with drug-susceptible TB; however, only a subset of strain families were found as the degree of drug resistance increased to MDR TB and XDR TB. The decrease in genetic diversity with increasing drug resistance suggests clonal expansion of MDR TB and XDR TB strains.

Research over the past decade on the drug-resistant TB epidemic in South Africa has uncovered regional differences in the molecular epidemiology of the disease (*25*). Our first report of 53 patients with XDR TB from Tugela Ferry showed that a single strain, the LAM4/KZN strain, accounted for >85% of cases (*28*); subsequent studies have confirmed that the LAM4/KZN strain predominates among drug-resistant isolates throughout KwaZulu-Natal Province (*16*,*25*,*29*). In contrast, studies from South Africa’s Western and Eastern Cape Provinces found that 54%–69% of MDR TB and XDR TB isolates belonged to the Beijing family (*25*,*27*). Strains causing drug-resistant TB cases from other provinces varied further; S, T1, and other families accounted for most cases (*20*,*25*). The reasons for these geographic differences remain uncertain. However, the findings from this study allow us to exclude the possibility that the difference exists because the LAM4/KZN strain is endemic among all TB cases and that its predominance among XDR TB cases is simply a reflection of its endemicity. In our study, we found that the LAM4/KZN strain accounted for only 6% of TB cases caused by drug-susceptible isolates and 27% of MDR TB cases. Rather, the same strains that are common among drug-resistant case-patients in other provinces (Beijing, S, T1) are also common among patients with drug-susceptible TB in KwaZulu-Natal.

These data allow us to consider potential causes for the emergence of drug resistance in KwaZulu-Natal at the beginning of the XDR TB epidemic. Drug-resistant TB occurs either as a result of acquired resistance—selection of resistance in individual patients due to incomplete or improper treatment—or through person-to-person transmission of drug-resistant strains. If acquired resistance were the predominant cause of drug-resistant TB cases, one would expect to find the same TB strains among MDR TB patients as in patients with drug-susceptible TB. By extension, acquired resistance would result in the same strains occurring among XDR TB patients as in MDR TB patients. In this study, however, most strain types prevalent in the drug-susceptible group were absent from the MDR TB strains, and most strains found in the MDR TB group were not found among XDR TB strains. Moreover, a few strains accounted for most MDR TB and XDR TB cases, suggesting clonal expansion. This study builds upon other evidence at the time of data collection (2005–2006), and more recently, which suggests that transmission of drug resistance played a major role in the MDR TB and XDR TB epidemic (*15*–*19*).

It has been hypothesized that certain TB strains have a greater ability than others to spread within a population (*22*,*23*). Numerous studies have suggested that the successful spread of certain Beijing strains may be due to their “hypervirulence,” which in part, involves a greater ability to evade host defenses (*23*). A few studies have also examined LAM4/KZN virulence and have shown they show greater adhesion to and invasion of human alveolar cells than other strains (*32*). LAM4/KZN may be more invasive than Beijing isolates while undergoing oxygen deprivation, a condition that mimics the environment in human granulomas (*33*). In addition, data examining the global population structure of *M. tuberculosis* suggest that certain TB lineages may have adapted over time to be more likely to cause disease in, and be transmitted among, specific sympatric human populations from particular geographic settings (*34*). It is unclear whether the geographic differences in the prevalence of drug-resistant strains in South Africa can be explained by such biological differences, or rather, are caused by local outbreaks related to patterns of human congregation and social mixing. Nonetheless, our study highlights the need for further studies to examine the host–pathogen interaction that may contribute to such geographic differences.

Regardless of whether the geographic differences found were caused by biological or social factors, implementing infection control policies and practices in congregate settings is essential. The clonal expansion seen in this study, along with countless other reports of drug-resistant TB transmission worldwide, highlight the major role that transmission plays in this epidemic (*10*–*13*,*19*). Implementation of rigorous infection control programs may curb transmission and avert a large proportion of future secondary cases (*35*). If such programs were implemented in settings such as KwaZulu-Natal, they may change the trajectory of the drug-resistant TB epidemic in a manner similar to what was seen in the United States in the 1990s (*36*). Unfortunately, to date, infection control programs have not been given priority in KwaZulu-Natal, and the incidence of XDR TB remains high (*37*).

Our study also showed that the association between strain type and mortality was attenuated when adjusted for degree of drug resistance, immune suppression, and extent of disease. This finding contrasts with studies that have shown a greater association of W/Beijing strains with disseminated disease and treatment failure (*38*). A recent study from the United States evaluated 4 lineages (East Asian, Euro-American, Indo-Oceanic and East-African Indian) and found an association between strain lineage and clinical site of disease, suggesting differences in pathogenicity and virulence of some strain types (*39*). However, that study did not include patients infected with *M. tuberculosis* from the LAM4/KZN strain family, nor was drug-resistance evaluated as a covariate.

This study is subject to certain limitations. First, the isolates in this study were obtained from patients with culture-positive TB disease for whom spoligotype results were available. Culture-taking practices vary across providers in KwaZulu-Natal and are not routinely obtained for all new patients suspected of having TB. Selection bias may have influenced the strain types found among each drug resistance group in this study. However, the decision to obtain a culture is determined without advance knowledge of strain types, so the differences observed between drug resistance groups are likely to reflect true group differences. Second, the isolates in this study were evaluated by using spoligotyping alone, which was used to assign strain families. More robust methods for defining lineages, such as single nucleotide polymorphism analysis or whole-genome sequencing, may have allowed for more refined assignment of strain families in this study. A recent study, however, directly compared spoligotyping with large sequence polymorphisms and single-nucleotide polymorphisms and found that spoligotyping could be used reliably to classify strain lineages in epidemiologic studies (*40*). In addition, the small sample size in each drug resistance group and known limitations of retrospectively obtained data from chart review may have prevented critical factors independently associated with mortality from being identified. Finally, the study took place at the time of a now, well-characterized, prolonged outbreak of XDR TB in Tugela Ferry. Although the limited genotypic diversity among XDR TB strains could be linked to this outbreak, it would not explain the small number of genotypes seen among isolates causing MDR TB cases at our site, nor the clonality seen among XDR TB isolates from other parts of KwaZulu-Natal (*16*) or in the Eastern Cape and Western Cape Provinces (*25*).

Despite these limitations, this study provides insight into genotypic diversity among drug-susceptible TB, MDR TB, and XDR TB strains in Tugela Ferry, an area with one of the highest rates of HIV and drug-resistant TB worldwide. The decrease in strain diversity with increasing drug resistance provides further evidence that the drug-resistant TB epidemic in KwaZulu-Natal was largely caused by transmission and clonal expansion of predominant strain types. This study also adds to the growing body of literature on strain geographic, clinical, and epidemiologic diversity and provides vital insights for generation of future hypothesis-driven studies of strain virulence and transmissibility. The study results underscore the need for expansion and implementation of sound policy and practices regarding TB/HIV integration and airborne infection control in community and health care facility settings throughout the world.

Technical Appendix Table showing spoligotype patterns (including binary format) of *Mycobacterium tuberculosis* isolates from patients in Tugela Ferry, KwaZulu-Natal Province, South Africa, 2005–2006.
