# Supplementary material for: Minimal Diversity of Drug-Resistant Mycobacterium tuberculosis Strains, South Africa
Source: Emerg Infect Dis. 2014 Mar;20(3):426–37. doi: 10.3201/eid2003.131083 (PMC3944869; doi:10.3201/eid2003.131083)
Supplement: Technical Appendix — Table showing spoligotype patterns (including binary format) of Mycobacterium tuberculosis isolates from patients in Tugela Ferry, KwaZulu-Natal Province, South Africa, 2005–2006. [file 13-1083-Techapp-s1.pdf]

Article DOI: http://dx.doi.org/10.3201/eid2003.131083

Minimal Diversity of Drug-Resistant *Mycobacterium tuberculosis* Strains, South Africa

Technical Appendix

Technical Apendix Table. Spoligotype patterns (including binary format) of *Mycobacterium tuberculosis* isolates from patients in Tugela Ferry, KwaZulu-Nata Province, South Africa, 2005–2006*,†

Lineage	Shared type	International family	Spoligotype pattern (binary format)	Octal code	DS-TB strains (n = 115)	MDR TB strains (n = 79)	XDR TB strains (n = 92)
Beijing	**1**	**Beijing**		**000000000003771**	**31**	**2**	**0**
LAM	4	LAM3/S		000000007760771	2	0	0
	33	LAM3		776177607760771	12	0	1
	42	LAM9		777777607760771	1	1	1
	**60**	**LAM4**		**777777607760731**	**7**	**21**	**82**
	211	LAM3		776137607760771	2	0	0
	811	LAM4		777777604060731	1	0	0
	1321	LAM1-LAM4		677777607760731	1	0	0
	1624	LAM3-LAM6		776177607560771	1	0	0
	1750	LAM4		777767607760731	0	1	0
S-family	**34**	**S**		**776377777760771**	**4**	**27**	**0**
	466	S		776377377760771	1	0	0
	831	S		776367777760771	1	2	0
T-family	37	T3		777377777760771	0	5	0
	39	T4-CEU1		777777347760471	1	0	0
	53	T1		777777777760771	10	6	4
	118	T2		777767777760771	1	0	0
	136	T1		777603405760471	0	0	1
	205	T1		737777777760771	2	0	0
	244	T1		777777777760601	2	1	0
	334	T1		577777777760771	1	0	0

Lineage	Shared type	International family	Spoligotype pattern (binary format)	Octal code	DS-TB strains (n = 115)	MDR TB strains (n = 79)	XDR TB strains (n = 92)
	358	T1		717777777760771	1	0	0
	719	T1		776177407760771	3	0	0
	766	T1		777761007760771	0	2	0
	879	T1		777767777760671	1	0	0
	926	T1		773777777760771	0	5	0
	1166	T1		777377777760771	0	1	1
	1547	T3		777727777760771	0	1	0
X-family	200	X3		700076777760700	1	0	0
	336	X1		777776777760731	0	0	1
	1751	X3		700066777760771	1	0	0
Haarlem	47	H1		777777774020771	2	0	0
	50	H3		777777777720771	2	0	0
	62	H1-variant1		777777774020731	1	1	0
	75	H3		777767777720771	1	0	0
	294	H3		577777777720771	1	0	0
Other	21	CAS1-Kili		703377400001771	3	0	0
	26	CAS1-Delhi		703777740003771	3	0	0
	71	EAI-undefined		776337777760771	1	0	0
	172	U		777777777740771	1	0	0
	374	U		777777776000771	1	0	0
	583	MANU2		777737777763771	1	1	0
	806	EAI1-SOM		757777777413731	1	0	0
	1092	CAS		702777740003771	3	0	0
